# Integrated active sensor system for real time vibration monitoring

**DOI:** 10.1038/srep16063

**Published:** 2015-11-05

**Authors:** Qijie Liang, Xiaoqin Yan, Xinqin Liao, Shiyao Cao, Shengnan Lu, Xin Zheng, Yue Zhang

**Affiliations:** 1 State Key Laboratory for Advanced Metals and Materials, School of Materials Science and Engineering, University of science and technology Beijing, Beijing, 100083, China; 2 Key Laboratory of New Energy Materials and Technologies, University of Science and Technology Beijing, Beijing, 100083, China

## Abstract

We report a self-powered, lightweight and cost-effective active sensor system for vibration monitoring with multiplexed operation based on contact electrification between sensor and detected objects. The as-fabricated sensor matrix is capable of monitoring and mapping the vibration state of large amounts of units. The monitoring contents include: on-off state, vibration frequency and vibration amplitude of each unit. The active sensor system delivers a detection range of 0–60 Hz, high accuracy (relative error below 0.42%), long-term stability (10000 cycles). On the time dimension, the sensor can provide the vibration process memory by recording the outputs of the sensor system in an extend period of time. Besides, the developed sensor system can realize detection under contact mode and non-contact mode. Its high performance is not sensitive to the shape or the conductivity of the detected object. With these features, the active sensor system has great potential in automatic control, remote operation, surveillance and security systems.

Vibration is one of the most common phenomena that exist in our daily life, which is generated as a result of mechanical disturbance with the sources of wind, sound, engine and many more. Detection of vibration is a useful sensor technology for monitoring machine operations^1^, warrant of security^2^ and prediction of disasters^3^. On the other hand, vibration developed in industrial machines can be one of the useful machine quantities to effectively monitor machining operations and detect the machine faults at an early state. Vibration monitoring of machinery as a part of maintenance system has received increased interest due to its potential advantages of reducing consequential damage, increasing machinery availability and performance, minimizing interruption to the production or supply system and ensuring the safe running of machine.

Information about the machine in the form of primary data will be obtained by the monitoring system and then through the use of signal processing and analysis technique, it is possible to provide the vital operation condition and diagnostic information of machine. Considerable efforts have been devoted to the development of a broad spectrum of vibration sensors based on acoustic^4^,^5^,^6^, optical^7^, thermal^8^, etc, sensing systems.

Almost all of the vibration sensors need to be driven by electric power, which is usually provided by batteries. The reliance on battery power will limit their development of sensor network with huge amounts of small sensor nodes due to the limited lifespan and periodical replacement of these batteries. Another limitation of these sensors is their limited capacity for multiplexed operation, because in many cases, the vibrations of a large amount of components need to be detected. Besides, some of these sensors are bulky, heavy and usually require other essential components, which further added complexity as well as cost. With these regards, a self-powered, small-sized, lightweight and cost-effective array vibration sensor system is greatly desirable for solving the above problems.

Here we develop a self-powered array sensor system for realizing multiplexed vibration monitoring. Based on contact electrification^9^,^10^,^11^,^12^,^13^,^14^,^15^,^16^ between the sensor and the detected objects, the designed array sensor system converts vibration of every detected unit into locally electric signals. Through processing and analyzing the output of the sensor, the vibration condition of every unit can be obtained, including: on-off state, vibration frequency and vibration amplitude of each unit. More importantly, the active array sensor system can record the vibration process of every unit, which is beneficial for monitoring the changing levels of vibration over time. Thus it is possible to present the history of the working condition of every unit and find the historical period that one unit is under abnormal state. The high performance won’t be influenced by the conductivity or form of the detected object. The sensor can perform normally under both contact-mode and non-contact mode. Last but not least, by connecting each pixel in the matrix with LED, the active sensor system can monitor the vibration condition of every unit more intuitive. Given such features as self-powered, lightweight, cost-effective, multiplexed operation, as well as the convenience of integrating with industrial machine, the active array sensor system presented in this work is a milestone in the development towards automatic control, remote operation, surveillance and security systems.

## Results

### Device structure

**Figure 1:**
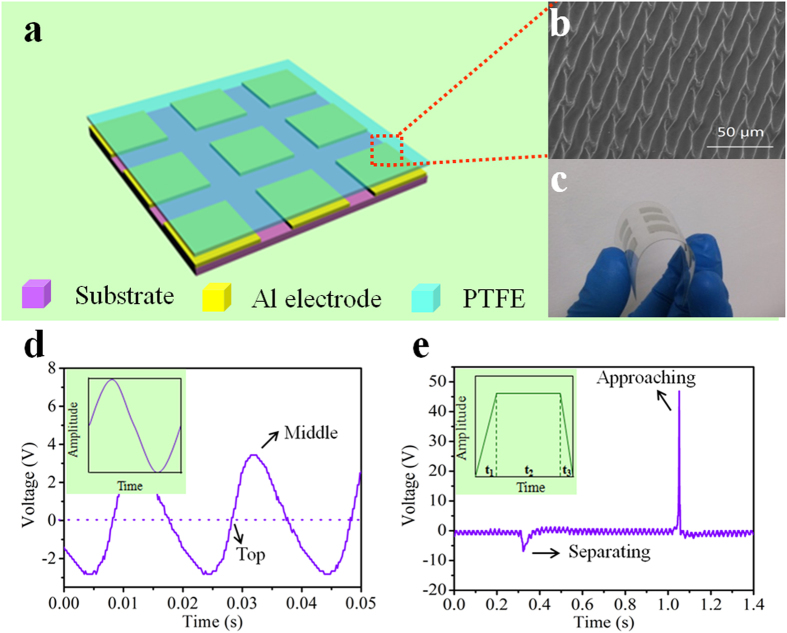
Structure illustration and typical electrical response of the active sensor system. (**a**) The schematic diagram of the active sensor system. (**b**) The SEM image of the PTFE film with patterned microstructure. (**c**) Photograph of a bent flexible device. Photograph taken by Qijie Liang. (**d**,**e**) Output voltage with different loading process.

The structure diagram of the fabricated self-powered array sensor is shown in Fig. 1a. It is structurally composed of an acrylic substrate, nine square Al electrodes and PTFE film. Figure 1b illustrates the scanning electron microscope (SEM) image of the PTFE film. The surface is uniformly covered by patterned microstructure, which can be used to increase the contact area and thus enhance the output of the sensor. The microstructure is obtained by applying a steel-wire screen onto the PTFE film with controlled pressure and the detailed fabrication process is presented in the experimental section. The active array sensor can be flexible by replacing the acrylic substrate by transparent polyethylene terephthalate (PET) substrate. Figure 1c displays the photograph of a flexible device. During the measurements, nine Al plates are employed in simulating the vibration of conductor and are attached to another acrylic sheet connected with a vibration output system. The vibration frequency and amplitude of the detected units are regulated by the function generator. The dimension of the fabricated array sensor is 5 cm * 5 cm.

### Typical electrical response on different loading process

Each pixel in the active sensor array can itself generate electric signals as a response to the vibration of every detected unit, which is the basic principle of the designed device acting as a self-powered vibration sensor. The electricity generation mechanism is based on the coupling between contact electrification and electrostatic induction^17^,^18^,^19^,^20^,^21^,^22^,^23^,^24^,^25^,^26^. The loading information of the vibration can be obtained by employing the detail of the output signal. Specifically, the output signal is relevant to the loading process of the vibration. For a sinusoidal loading process (inset of Fig. 1d), the output voltage is shown in Fig. 1d, which is also a sinusoidal shape. The output becomes 0 when the vibrating unit reaches the highest position, while the output achieves biggest value when the position is middle. For a loading process of the inset of Fig. 1e, the output is displayed in Fig. 1e. The output voltage has an alternating behavior with asymmetrical amplitude. The larger peaks correspond to the process in which the vibrating unit approaches the sensor fast, while the smaller one is generated as the unit leaves the sensor slowly.

### On-off state monitoring of nine vibrating units

**Figure 2:**
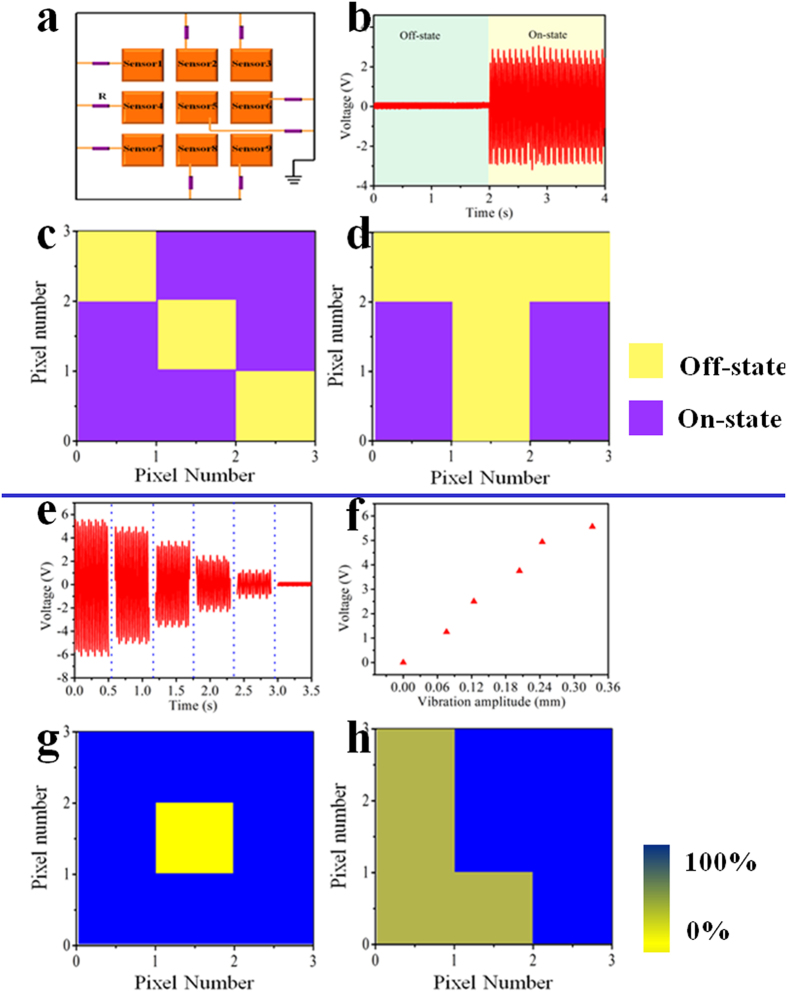
On-off state monitoring and working amplitude monitoring of nine vibrating units. (**a**) Schematic diagram of the fabricated array sensor. (**b**) The output of a single unit under off-state and on-state. (**c**,**d**) Measured mapping figures with different amounts of units in off-state. (**e**) The output of a vibrating unit with different vibration amplitudes. (**f**) Dependence of the output voltage on the vibration amplitude. (**g**,**h**) Measured mapping figures with the working amplitudes of different amounts of units deviated from normal set value.

The schematic diagram of the active array sensor is depicted in Fig. 2a. All the units in the array work in single-electrode mode and the electrodes are all connected to ground. As mentioned above, the array sensor will generate electric signals as response to the vibration of every unit, so the first monitoring content is the working state of every detected unit, which can be expressed as on-state and off-state. As shown in Fig. 2b, there will be no output when one unit does not vibrate, which illustrates that the unit is not working or in off-state. Once the detected unit vibrates, the corresponding sensor will generate outputs. The output voltage signals of nine units in the array sensor can be recorded as a mapping figure. The working state of nine detected units can be obtained by analysis of the mapping figures by addressing and monitoring the output voltage signals of every unit in the array sensor system. Figure 2c,d display the measured mapping figures with different amounts of detected units in off-state (corresponding output of each pixel is in Figure S1-S3). It can be found that the corresponding units represented by yellow region in the mapping figures are in off-state, which benefits the rapid diagnosis of the faults in the detected units. This is of great practical significance in equipment operation and manufacturing industry.

### Vibration amplitude monitoring of nine vibrating units

When the Al plate is brought into contact with PTFE, charge transfer will occur at the contact interface. According to the triboelectric series^27^,^28^, electrons are injected from Al plate to the PTFE film, with the PTFE film negatively charged and the Al plate positively charged. It is worth noting that the surface charges of the PTFE can be retained for an extended period of time and won’t dissipate and that is the reason that the vibration sensor can perform well under non-contact mode, which has been reported in our previous work. The surface charge density σ_1_ on the Al electrode can be given as follows^29^:


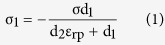


where σ, d_1_ are the surface charge density and thickness of PTFE, respectively. ε_rp_ is the relative permittivity of PTFE and d_2_ is the distance between the detected unit and PTFE surface. The change of distance will result in the variation of the surface charge density σ_1_ of the Al electrode, which means the vibration amplitude will influence the output of the vibration sensor. This is the basic principle of the array sensor for monitoring the vibration amplitude of every detected unit. Figure 2e displays the output voltages of one pixel with the vibration amplitude decreasing from the maximum value to zero. The summarized relationship between the vibration amplitude and the output voltage is shown in Fig. 2f, from which we can find that the output voltages vary almost linearly with the change of the vibration amplitudes. For a fixed monitored vibrating component, it usually performs normally with settled vibration amplitude. The self-powered array sensor system can monitor the change of vibration amplitude of every vibrating unit and estimate that whether the working condition of each detected unit is deviated from formal state. Here we set vibration amplitude of 0.34 mm to be the formal value, and then the deviation of the vibration amplitude from every unit can be detected. Figure 2g,h show the measured mapping figures of the variation of vibration amplitudes of nine detected units (corresponding output of each pixel is in Figure S4-S6). When detected units are vibrating with formal or settled amplitude, the voltage outputs from all the pixels are at background level, which is defined as 100% amplitude of settled value. From the mapping figures of Fig. 2g,h, the outputs of one and four pixels in the array deviate from normal value, demonstrating that the vibration amplitudes of the corresponding detected units are 0%, 56–60% of settled value, respectively.

### Vibration frequency monitoring of nine vibrating units

**Figure 3:**
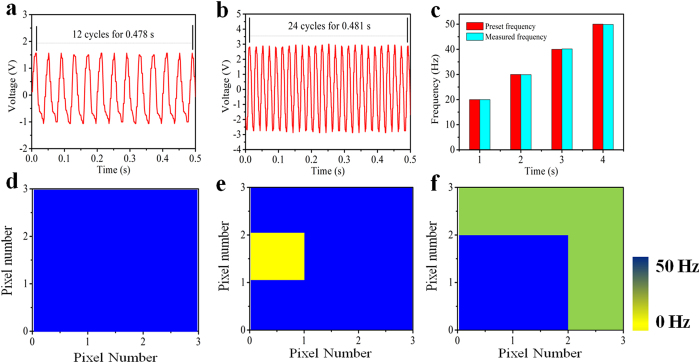
Working frequency monitoring of nine detected units. (**a**) The output of a vibrating unit with preset frequency of 25 Hz. (**b**) The output of a vibrating unit with preset frequency of 50 Hz. (**c**) The comparison between the preset vibration frequency and measured frequency. (**d**,**f**) Measured mapping figures with the working frequency of different amounts of units deviated from normal set value of 50 Hz.

The vibration frequency of every unit is a crucial indicator for monitoring the condition of vibrating components. The array sensor system developed in this work can detect the vibration frequency of each unit with high accuracy. Figure 3a,b show the output voltage of one pixel with settled vibration frequency of 25 and 50 Hz. The calculated frequencies are 25.10 and 49.90 Hz, respectively, with the relative errors of 0.42% and 0.21%. Figure 3c shows the comparison between the preset frequency and the measured frequency, which confirms the high accuracy of the vibration sensor. For a fixed monitored vibration component, it usually functions well with a settled vibration frequency. The self-powered array sensor system can monitor the vibration frequency of every vibration unit and estimate that whether it is deviated from formal state. The measured mapping figure of vibration frequency of nine units with settled frequency of 50 Hz is displayed in Fig. 3d. If we set a formal working frequency of the entire vibrating units to be 50 Hz, the deviation of the vibration frequency generated from per unit can be detected in real time. From the mapping figure of Fig. 3e, the output voltage of a pixel in the array is 0, which means the vibration frequency of the corresponding unit is 0 Hz. Figure 3f depicts that the vibration frequencies of 5 detected units are 30 Hz, deviating from normal state(corresponding output of each pixel is in Figure S7–S9).

### Vibration history monitoring on the time dimension

**Figure 4:**
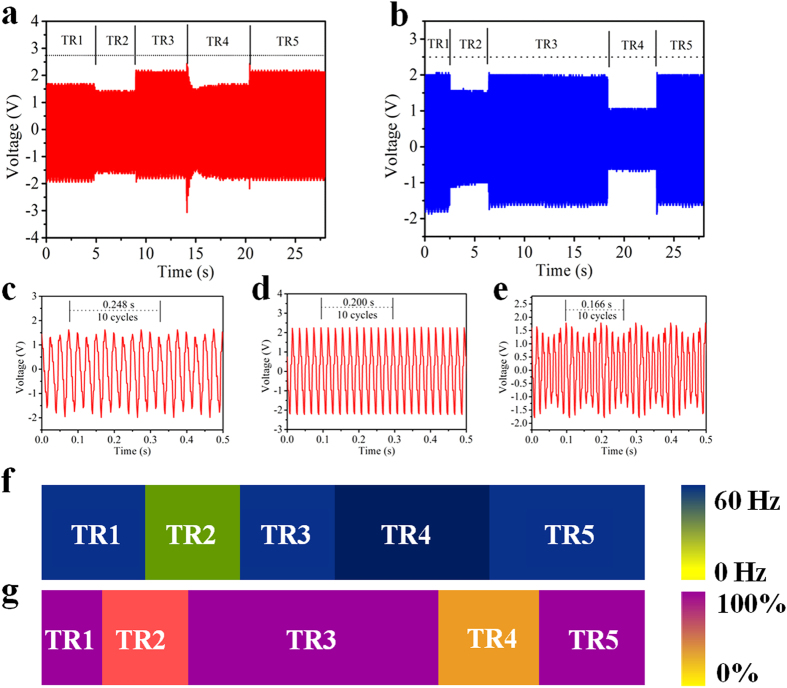
Working history monitoring on the time dimension. (**a**) The output electric signal of one vibrating unit for vibration frequency history monitoring in an extend period of time. (**b**) The output electric signal of one vibrating unit for vibration amplitude history monitoring. (**c**–**e**) The detailed outputs corresponding to TR2, TR3, TR4 in (**a**). (**f**) Measured mapping figure of a vibrating unit with working frequency deviated from settled value in some specific historical period. (**g**) Measured mapping figure of a vibrating unit with working amplitude deviated from settled value in some specific historical period.

For continuously vibrating components, record the vibration process of every unit is a fascinating issue, because the historical working condition of each unit can be obtained by analyzing the vibration process. The self-powered array sensor system can realize the recording of vibration process from all the components. Figure 4a,b show the output voltage signals of one unit in the array in an extend period of time. The time axis is divided into five time region (TR) due to the characteristic of the output signals for presenting the vibration state of the unit in different time region. The details of TR2, TR3 and TR4 section in Fig. 4a are displayed in Fig. 4c–e. The vibration frequencies in TR1, TR3 and TR5 are 50 Hz. The vibration frequencies in TR2 and TR4 are about 40 Hz, 60 Hz, respectively. Figure 4f shows the measured mapping figures for monitoring the vibrating frequencies of five time region in Fig. 4a. If we assume 50 Hz to be the formal vibration frequency, the vibration frequency represented by light green of TR2 and dark green of TR4 have deviated from normal value in two historical periods of TR2 and TR4. Figure 4b, g show the output signals and measured mapping figures for monitoring the vibration amplitude in TR1-TR5 with the vibration frequency of 50 Hz. Assume the vibration amplitude of TR1, TR3, TR5 to be formal value, then the vibration amplitudes represented by TR2 and TR4 in Fig. 4g were not in normal value in the historical periods of TR2 and TR4, which are 78.1%, 53.1% of the maximum value. In addition to the monitoring of vibration on large scale on the spatial dimension, the designed sensor system can monitor the vibration process of every unit on the time dimension.

### Demonstration of the wide range of applications

**Figure 5:**
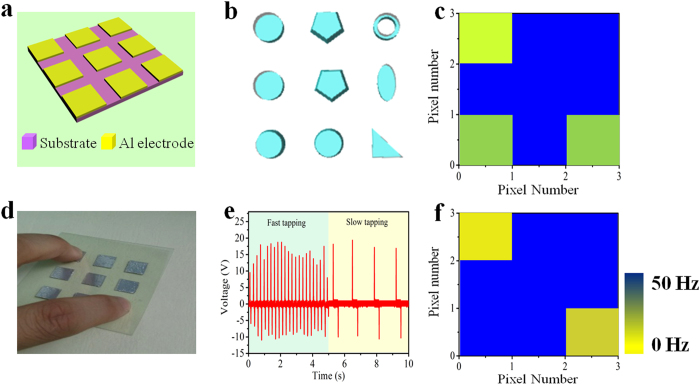
Demonstration of wide range of applications. (**a**) The schematic diagram of the active array sensor. (**b**) Illustration of the simulated shapes. (**c**) Measured mapping figure with 3 units vibrating under different frequencies. (**d**) Photograph of the matrix touched by two fingers. Photograph taken by Qijie Liang. (**e**) The output of a single unit with one finger tapping on it fast and slowly. (**f**) Measured mapping figure when two pixels in the matrix in (**d**) were knocked by two fingers under different frequency.

To demonstrate the wider range of potential applications of the active sensor system, we demonstrate that the sensor system can not only be utilized to monitor the vibration condition of conductive and regular-shaped units, but also employed in detecting the vibration condition of insulated units and units in different shapes. For detecting the vibration of insulated units, the structure of the sensor can be simpler, as depicted in Fig. 5a. Figure 5b displays the schematic diagram of the simulated detected units with different shape. For simulating objects with different shapes, the most important issue is the bottom shape that contact with the sensor. So the PTFE film was tailored to different shapes. In essence, the tailored PTFE film with fixed thickness is capable of simulating vibrating objects in different shapes. The measured mapping figures for monitoring the vibration condition of insulators are shown in Fig. 5c. The vibration frequencies of 3 units are 20, 25, 25 Hz and 50 Hz for the remaining units (corresponding output of each pixel is in Figure S10-S11). We also demonstrate that the active array sensor can detect the tapping of fingers. The photograph of the matrix touched by two fingers is shown in Fig. 5d. Figure 5e illustrates the output of a pixel in the array with one finger tapping fast and slowly, respectively. The Measured mapping figure for monitoring vibration frequency of the fingers is displayed in Fig. 5f, from which the tapping frequency of the fingers can be obtained, that is 0.77, 6.31 Hz, respectively(corresponding outputs is in Figure S12).

### Intuitively illustrates working condition of nine units with LEDs as indicators

**Figure 6:**
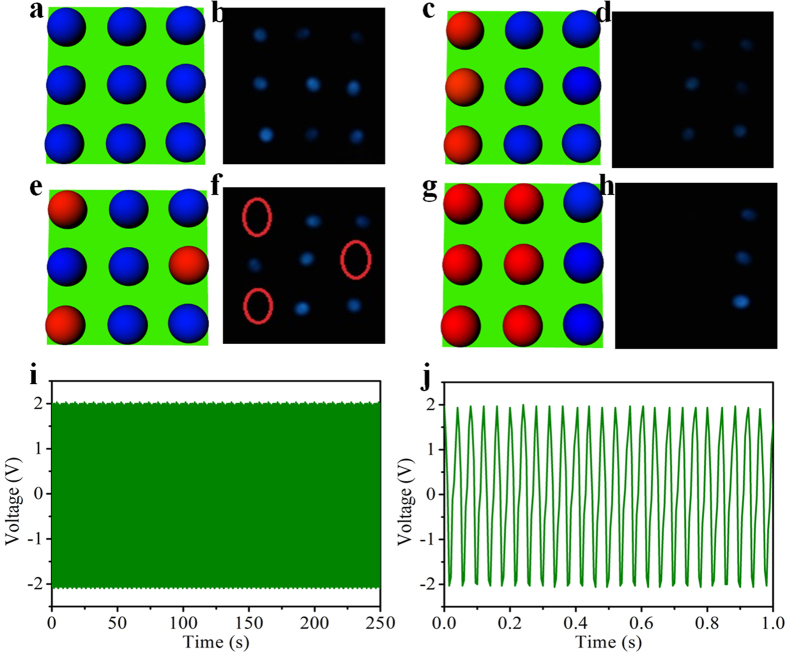
Intuitively illustrates working condition of nine units with LEDs as indicators. (**a**,**c**,**e**,**g**) The schematic diagram that every LED is connected with one pixel in the matrix. (**b**,**d**,**f**,**h**) The snapshots that different amount of LEDs connected to the sensor system extinguished. (**e**–**f**) The stability test of the self-powered sensor system. Photograph taken by Qijie Liang.

We also demonstrate that the self-powered array sensor system can monitor the vibration condition of each detected unit in real time with light-emitting diodes (LEDs) as indicators. The energy harvested from the vibration of every unit can drive LED instantaneously, which provides the possibility of presenting the working condition of each unit visually and directly. Figure 6a,c,e,g show the schematic diagrams of LEDs and every LED is connected to one pixel in the sensor array and the measurement results are displayed in Fig. 6b,d,f,h. As shown in Fig. 6b, all the LEDs are light up when the vibrating units are in normal state. Figure 6d,f,h are the snapshots that different amount of detected units are in abnormal state or off-state. For example, three LEDs in Fig. 6f are extinguished, which illustrates that the corresponding units connected to the LEDs represented by red hemispheres in Fig. 6e are suffering from faults. This will immediately remind us about that the units in trouble need to be maintained, which can give vital diagnosis information to operator before the equipment catastrophically fails. The stability of the array vibration sensor plays a key role in the practical application for vibration monitoring. The robustness of the sensor is also investigated and the results are shown in Fig. 6I,j. No attenuation of the electric output is observed after more than 10000 cycles, which verifies the excellent stability of the sensor.

## Discussions

Detection of vibration is a useful sensor technology for monitoring machine operations, warrant of security and prediction of disasters. Traditional passive sensor like laser technology that usually used in vibration detection has its limitations in many applications due to its nature such as, the necessity of external power, bulky, heavy and the lack of capacity of multiplexed operation. On the other hand, contact electrification is a universal phenomenon that charges are generated when two materials contact with each other. Recently, this effect is applied to design and fabricate triboelectric nanogenerator (TENG), presenting to be powerful, simple and reliable for harvesting ambient mechanical energy. Considering these factors, we design an integrated active sensor array based on contact electrification for real time monitoring vibration. It has several advantages over conventional passive vibration sensor, as is elaborated below.

Firstly, the active sensor array has no power consumption and can be further combined with the energy scavenging functionality for self-powered vibration monitoring. Besides, the functionality of multiplexed operation makes the sensor capable of monitoring vibration of large-scale units. Furthermore, the active array sensor can record the vibration process of detected units, facilitating the analysis of the corresponding working condition in an extend period of time. Last but not least, the fabrication process of the sensor array is straightforward and extremely low cost can be achieved.

The matrix in this work consists of 9 units (3 * 3) with the working dimension of each pixel of 1 cm * 1cm. To increase the range of the active sensor system for vibration monitoring, future efforts will be focused on: (a) the integrating of more units for real time monitoring the vibration of large amount of vibrating units. (b) the miniaturization of the pixel size to achieve higher spatial resolution. (c) the integration of the active sensor system on fully flexible substrate.

In summary, an active sensor system for vibration monitoring was demonstrated on the basis of contact electrification. The sensor can realize multiplexed operation to monitor the vibration of large amounts of units in real time including: on-off state, vibration frequency, vibration amplitude. Vibration process memory can be obtained by recording the outputs of sensor system in an extend period of time, which is beneficial for monitoring the changing levels of vibration over time. The active sensor system is capable of presenting a high performance regardless of the conductivity and form of detected objects. Given such features as self-powered, the ability of multiplexed operation, vibration process memory as well as lightweight, cost-effective, the active sensor system has potential applications in automatic control, remote operation, surveillance and security systems.

## Methods

### Fabrication of the active sensor system for detecting the vibration of conductors

To fabricate the sensor, a square-shaped acrylic sheet with a dimension of 5 cm by 5 cm by 1 mm was cut as a substrate. Nine pieces of aluminum foil (1cm * 1 cm) were tailored as the electrodes of the sensor, and securely attached them on the substrate with the distance of electrodes to be 1 cm and the conducting copper wires were connected to every electrode for subsequent electric measurements. Then, the Steel-Wire Screen was applied to the PTFE film (5 cm * 5 cm * 100 μm) with the pressure of 15 Mpa for ten minutes to obtain the patterned microstructure on the surface of the PTFE film. Subsequently, the acquired PTFE film was washed with acetone, isopropyl and alcohol for 10 minutes, respectively, and finally adhered it onto the prepared substrate with patterned Al electrodes.

### Fabrication of the active sensor system for detecting the vibration of insulators

The preparation of the substrate and Al electrodes were the same like the above steps. Attach the electrodes on the substrate and connect to the copper wires. For simulating the vibration of the insulators, nine units of PTFE slices were adhered onto the same substrate with that in the device.

### Characterization

During the measurements, the device was fastened on a stationary stage, and the detected vibrating objects were connected to a vibration output system with the vibration frequency and amplitude regulated by a function generator. A digital oscilloscope (DS4052, RIGOL) was applied to measure the output of the sensor. All the measurements were carried out in the ambient environment at room temperature.

## Additional Information

**How to cite this article**: Liang, Q. *et al.* Integrated active sensor system for real time vibration monitoring. *Sci. Rep.* **5**, 16063; doi: 10.1038/srep16063 (2015).

## Supplementary Material

Supporting Information
