# Supplementary material for: Integrated active sensor system for real time vibration monitoring
Source: Sci Rep. 2015 Nov 5;5:16063. doi: 10.1038/srep16063 (PMC4633595; doi:10.1038/srep16063)
Supplement: Supporting Information [file srep16063-s1.pdf]

Integrated active sensor system for real time vibration monitoring

Qijie Liang[1], Xiaoqin Yan[1,*], Xinqin Liao[1], Shiyao Cao[1], Shengnan Lu[1], Xin Zheng[1], Yue Zhang[1,2,*]

[1]State Key Laboratory for Advanced Metals and Materials, School of Materials Science and Engineering, University of science and technology Beijing, Beijing, 100083, China

[2] Key Laboratory of New Energy Materials and Technologies, University of Science and Technology Beijing, Beijing, 100083, China

* Corresponding author: Key Laboratory of New Energy Materials and Technologies, University of science and technology Beijing, Beijing, 100083, China.

E-mail: yuezhang@ustb.edu.cn (Y. Zhang), xqyan@mater.ustb.edu.cn (X. Yan).

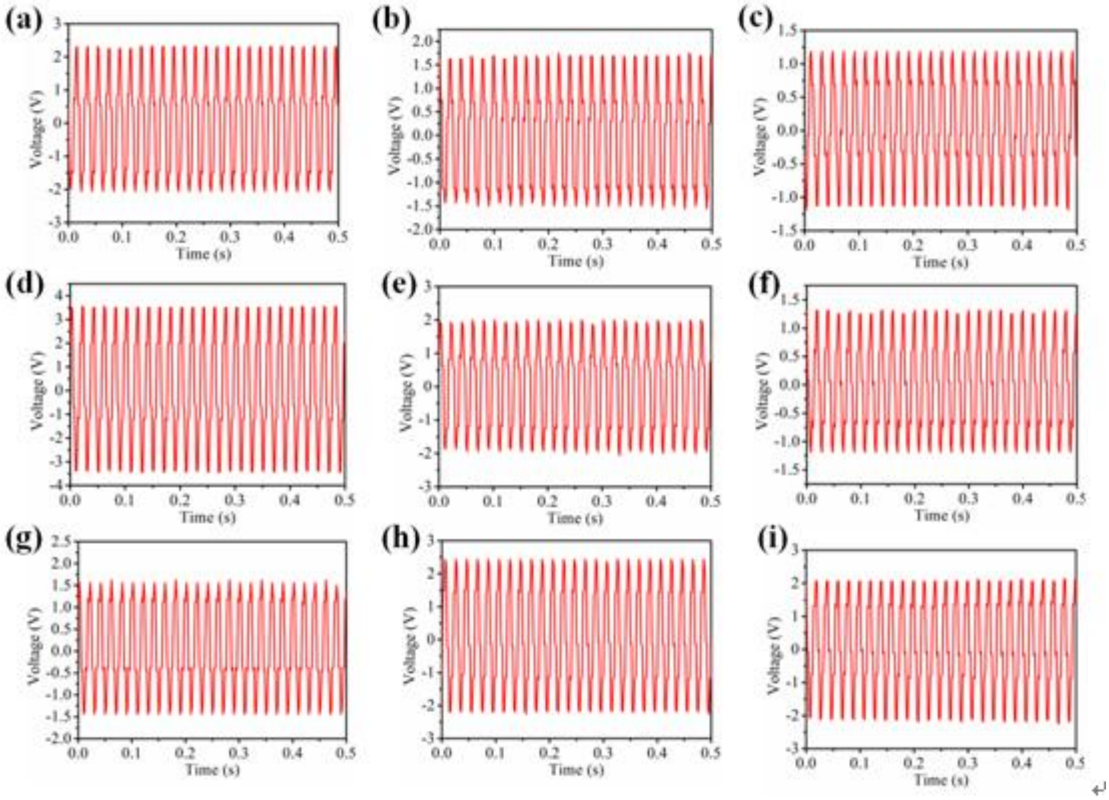

Figure S1. The outputs of nine pixels in the sensor array with all detected units in on-state.

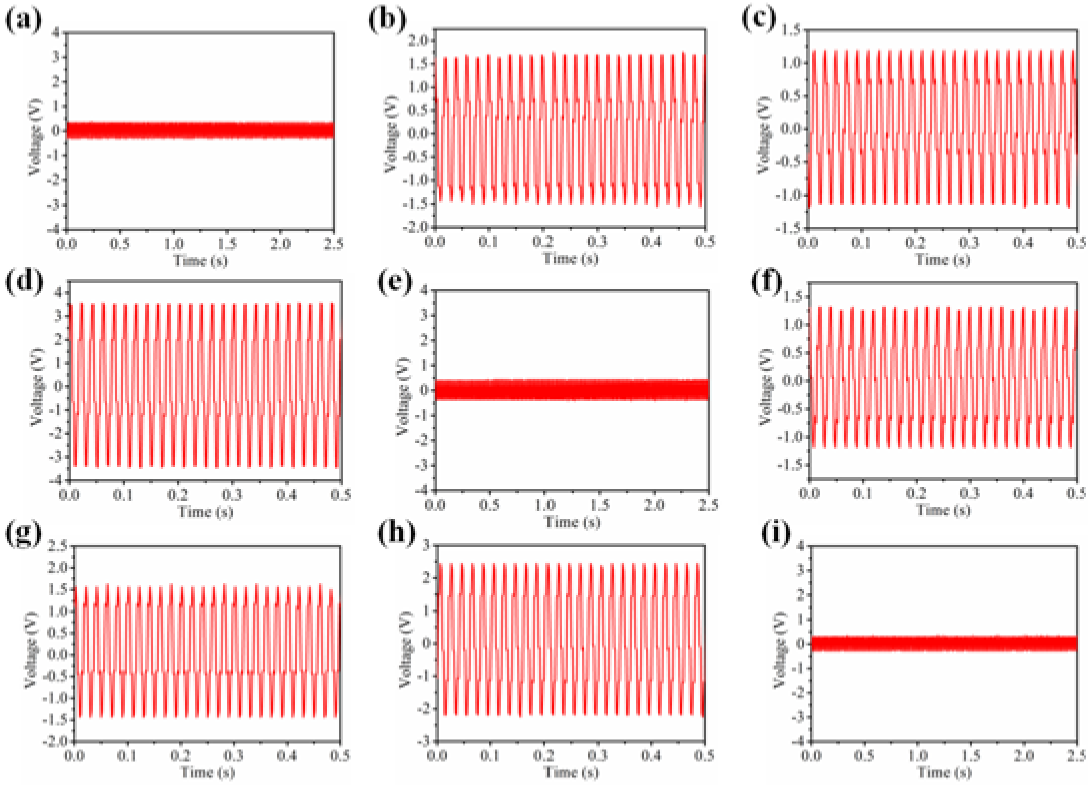

Figure S2. The outputs of nine pixels in the sensor array with 3 units in off-state.

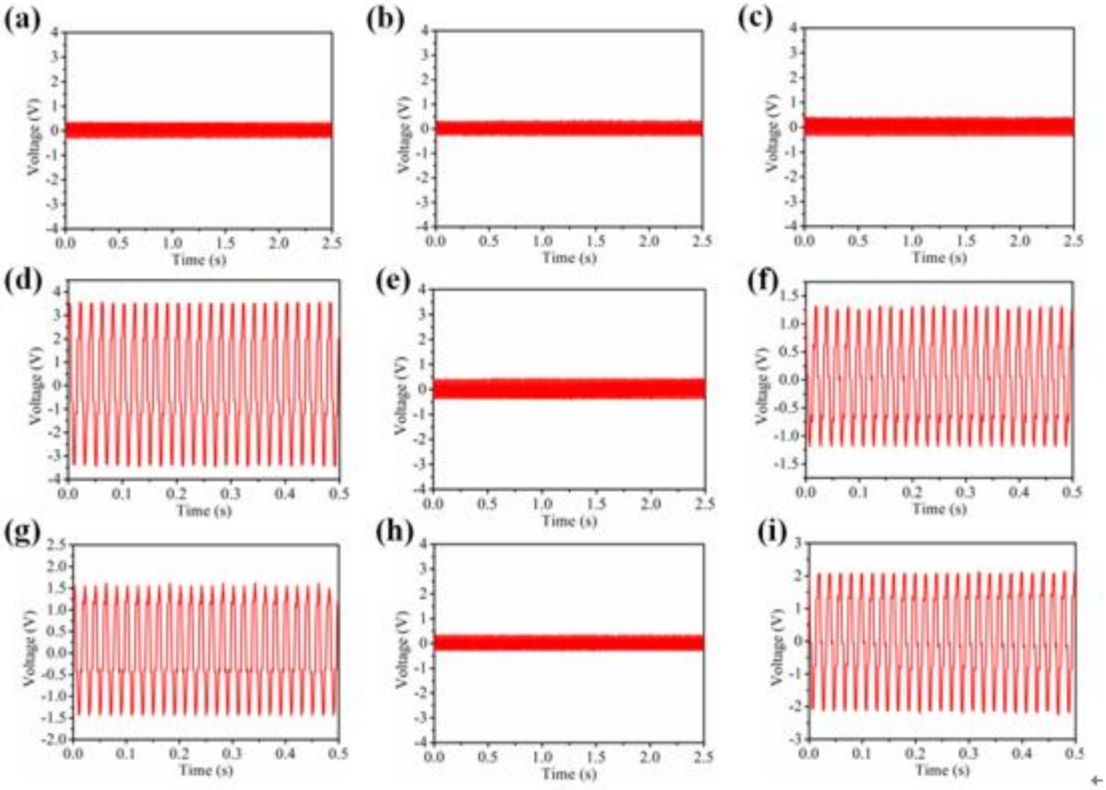

Figure S3. The outputs of nine pixels in the sensor array with 5 units in off-state.

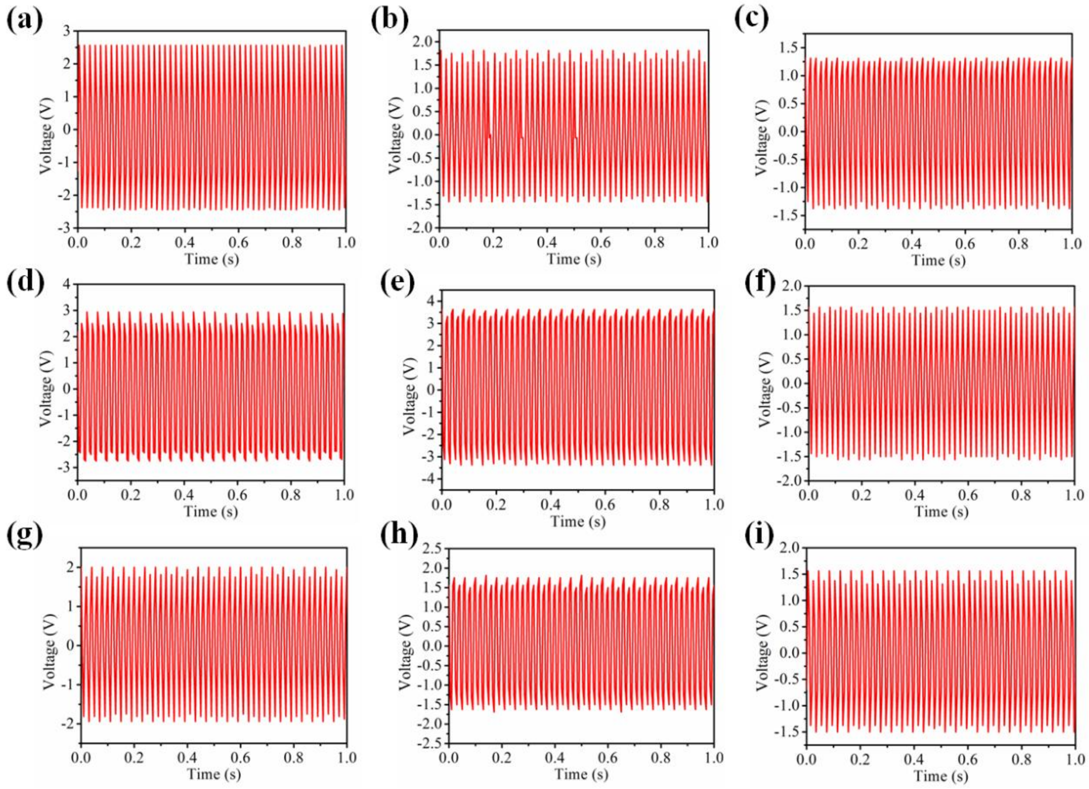

Figure S4. The outputs of nine pixels in the sensor array with vibration amplitude of normal set value.

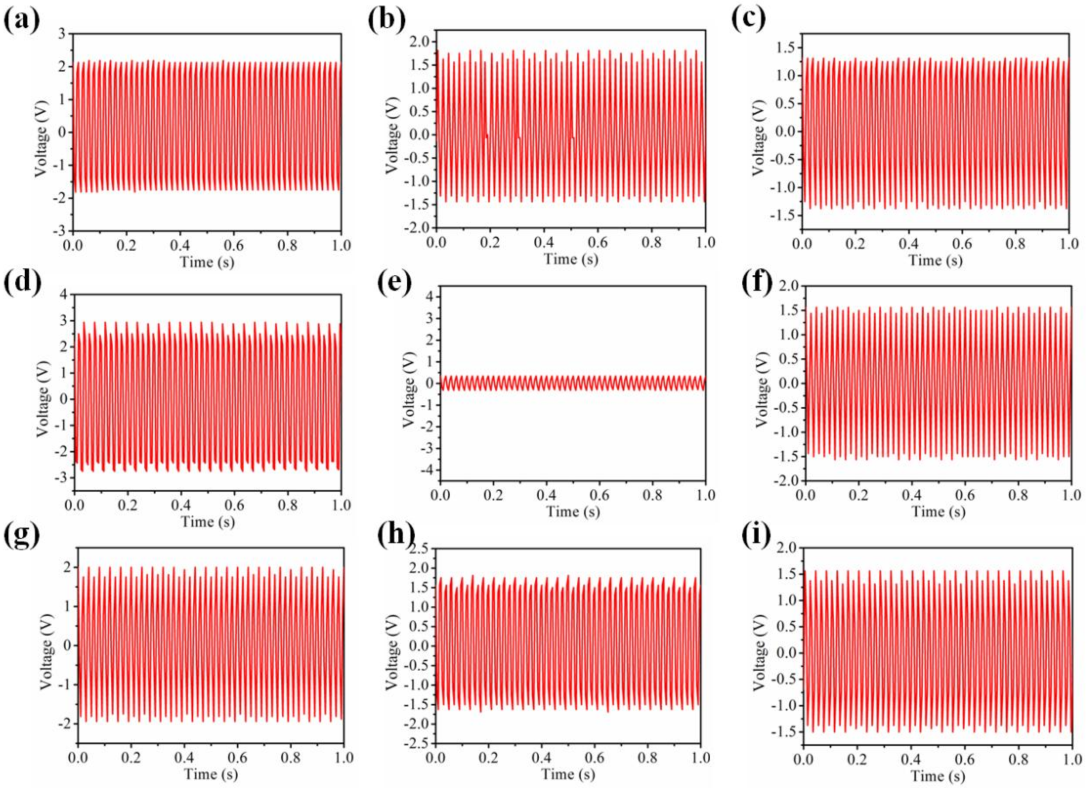

Figure S5. The outputs of nine pixels in the sensor array with vibration amplitude of 1 unit deviated from normal set value.

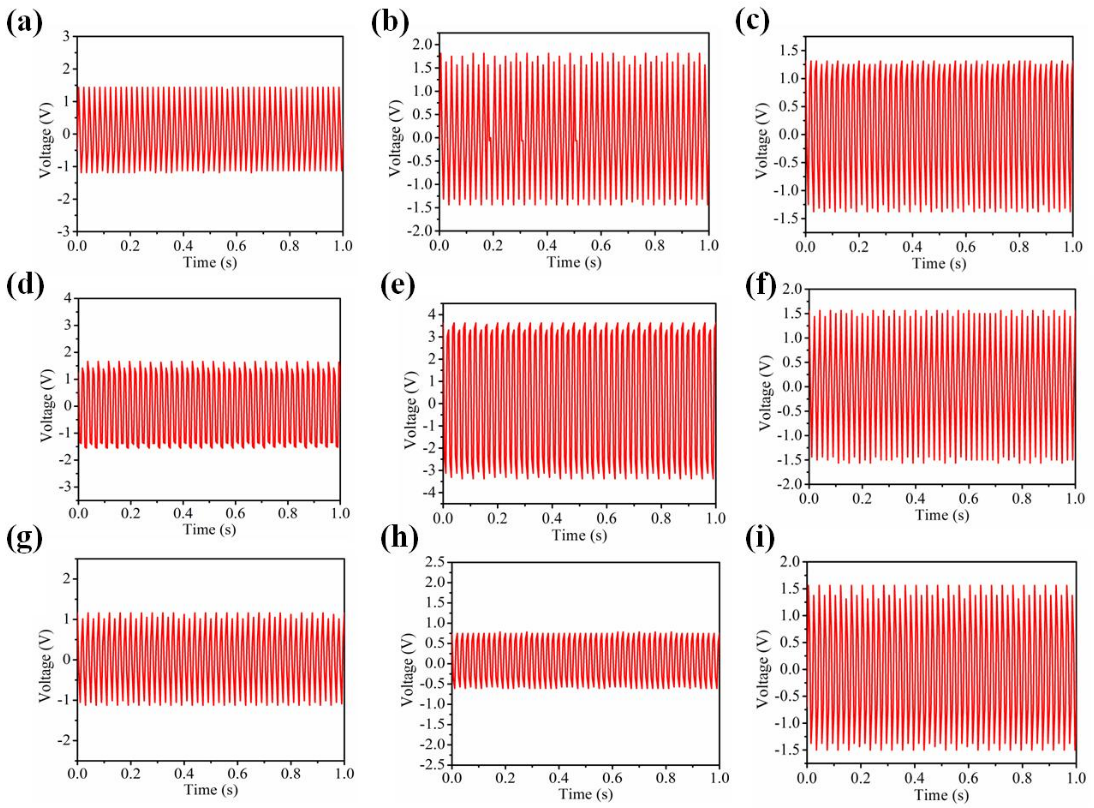

Figure S6. The outputs of nine pixels in the sensor array with vibration amplitude of 4 units deviated from normal set value.

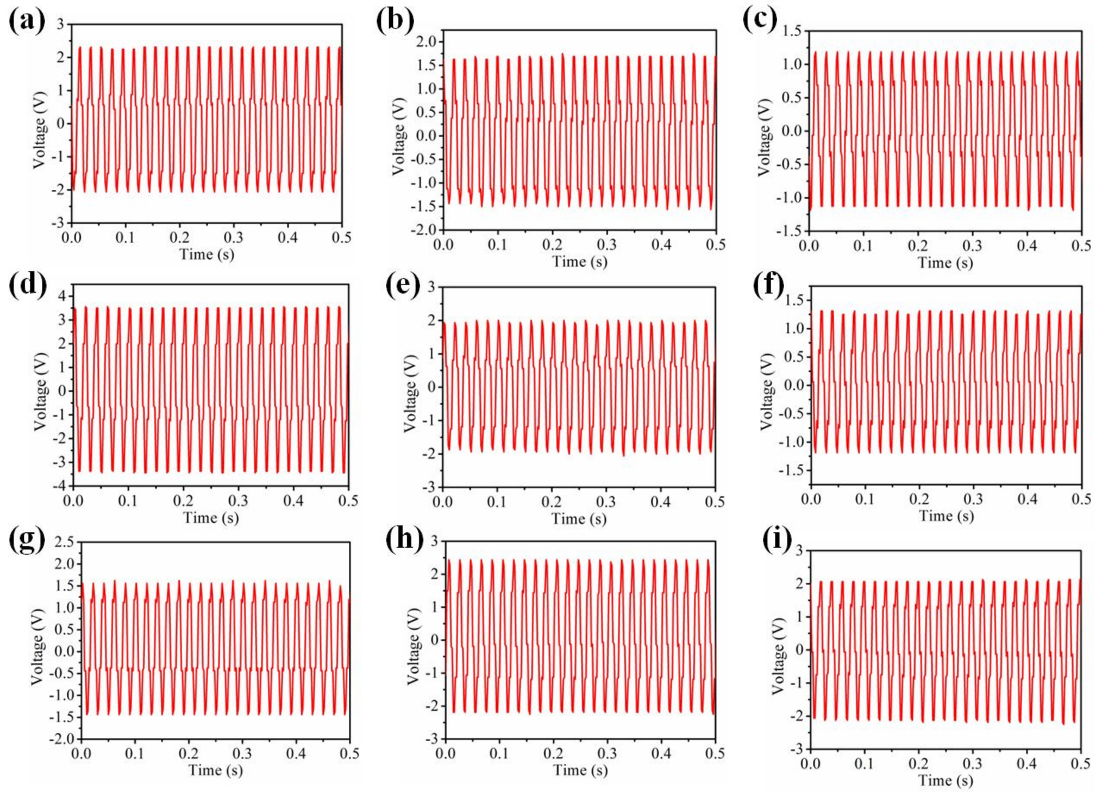

Figure S7. The outputs of nine pixels in the sensor array with vibration frequency of all units to be 50 Hz.

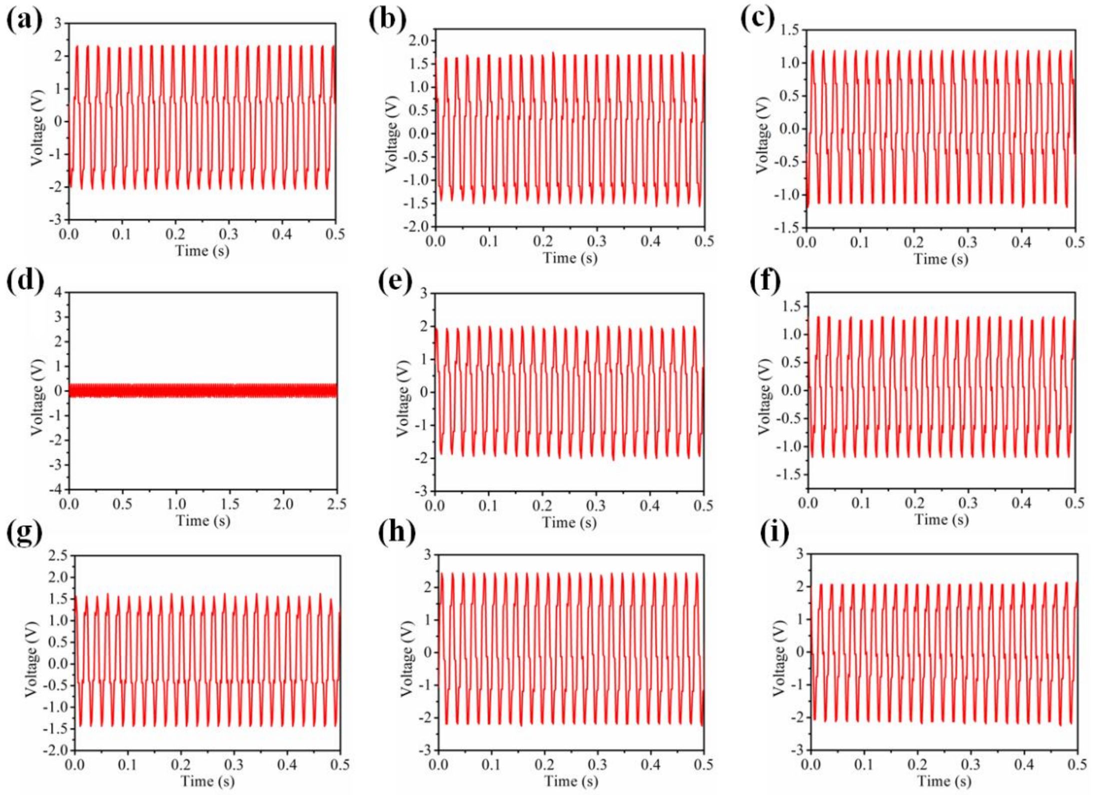

Figure S8. The outputs of nine pixels in the sensor array with vibration frequency of 1 unit deviated from normal set value of 50 Hz.

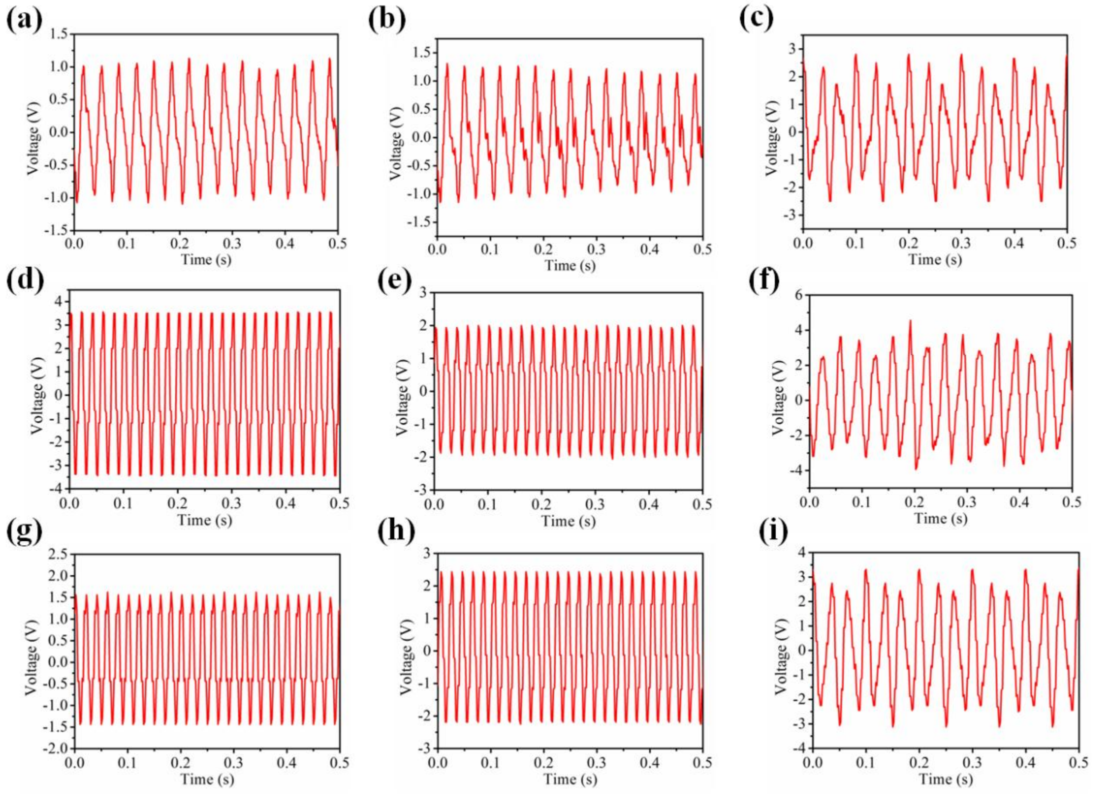

Figure S9. The outputs of nine pixels in the sensor array with vibration frequency of 5 units deviated from normal set value of 50 Hz.

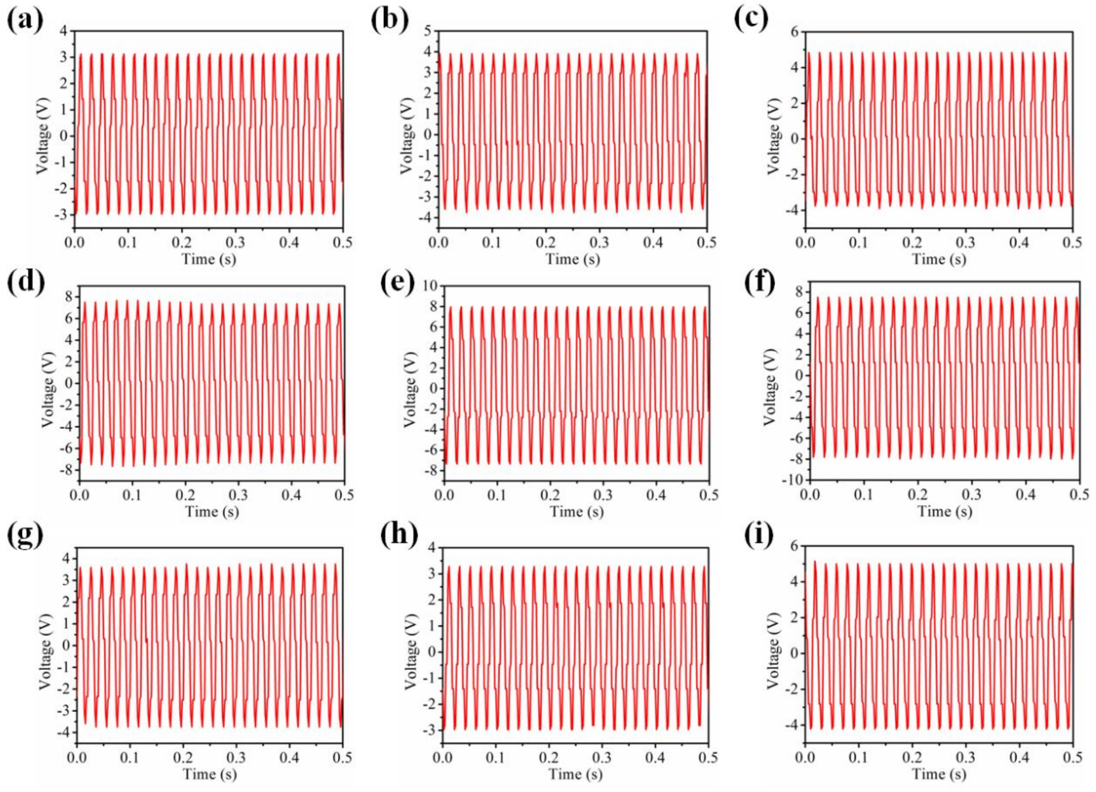

Figure S10. The outputs of nine pixels in the sensor array with vibration frequency of all units to be 50 Hz.

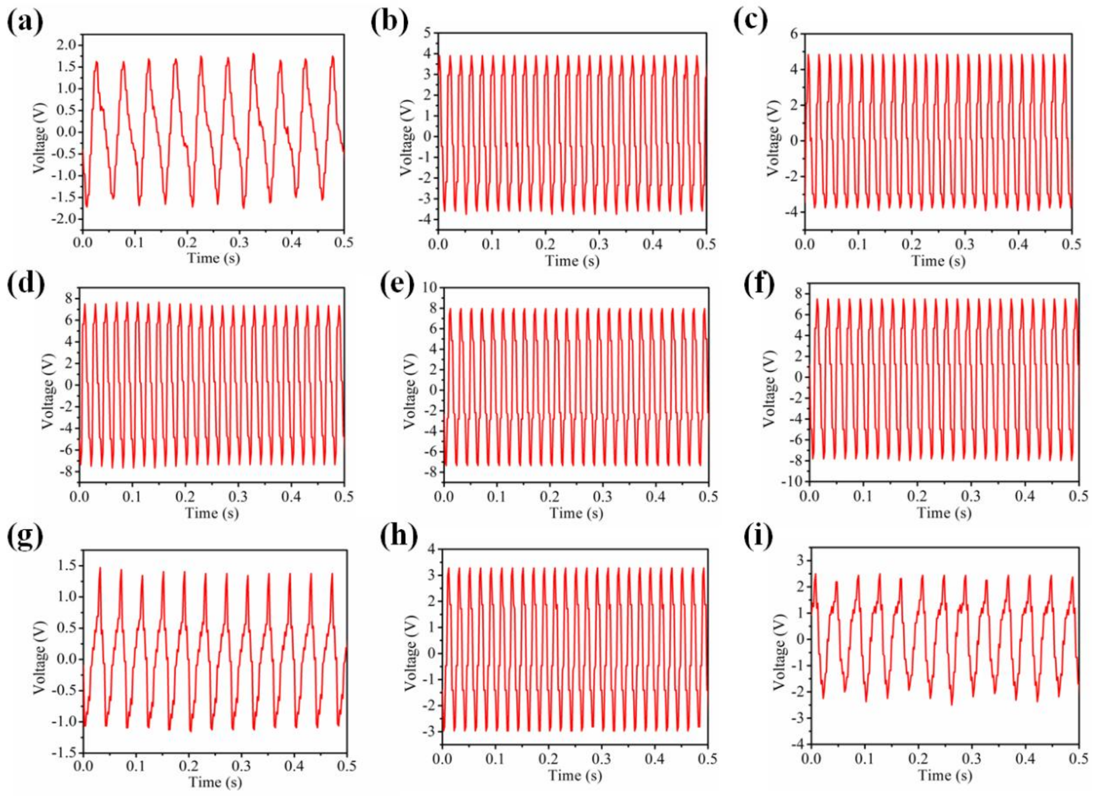

Figure S11. The outputs of nine pixels in the sensor array with vibration frequency of 3 units deviated from normal set value of 50 Hz.

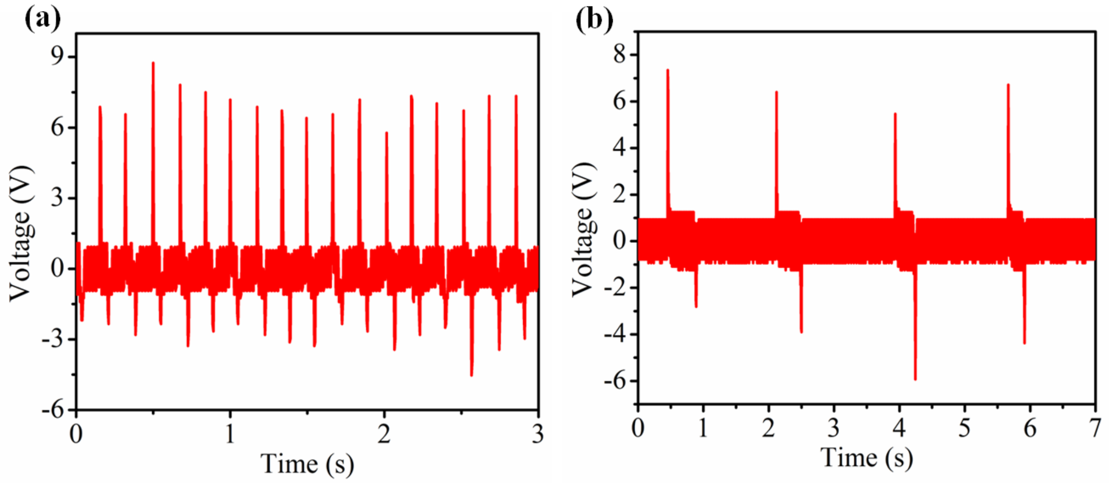

Figure S12. The outputs of 2 pixels in the sensor array when two pixels in the matrix were knocked by two fingers under different frequency.
